# Plasmonic Nano-Polymersomes for Pulsed Laser-Triggered Intracellular Cargo Release

**DOI:** 10.1021/acsomega.6c08089

**Published:** 2026-09-16

**Authors:** Sebastian A. Zmijewski, Jesse Cecero, Sean M. O’Malley, Eric A. Klein, Julianne C. Griepenburg

**Affiliations:** † 15636 Rutgers University-Camden, Center for Computational and Integrative Biology, Camden, New Jersey 08102, United States; ‡ 15636 Rutgers University-Camden, Department of Physics, Camden, New Jersey 08102, United States; § Rutgers University-Camden, Department of Biology, Camden, New Jersey 08102, United States

## Abstract

Polymersomes are synthetic vesicles capable of encapsulating both hydrophilic and hydrophobic cargo, making them attractive candidates for drug delivery applications. The inherent stability of polymer vesicles, such as poly­(butadiene)_33_-*b*-poly­(ethylene oxide)_20_ polymersomes, is advantageous for cargo retention, but can also make controlled cargo release a challenge. In this work, nano-polymersomes containing membrane-incorporated gold nanoparticles (AuNPs) were investigated as a pulsed laser-responsive delivery platform for intracellular cargo release. Hydrophobic AuNPs embedded within the membrane serve as plasmonic photosensitizers, rendering the vesicles responsive to 532 nm picosecond and nanosecond laser irradiation. To evaluate their utility for intracellular cargo release, doxorubicin was encapsulated within the aqueous core of the polymersomes. Polymersomes were evaluated for stability in common cell culture buffers and demonstrated efficient internalization with negligible cytotoxicity in NIH 3T3 cells. Following irradiation, doxorubicin-loaded polymersomes produced a significant reduction in cell viability, whereas control groups exhibited minimal toxicity. These findings demonstrate the potential of AuNP-polymersomes as candidates for externally triggered drug delivery vehicles for laser-mediated intracellular cargo release.

## Graphical abstract

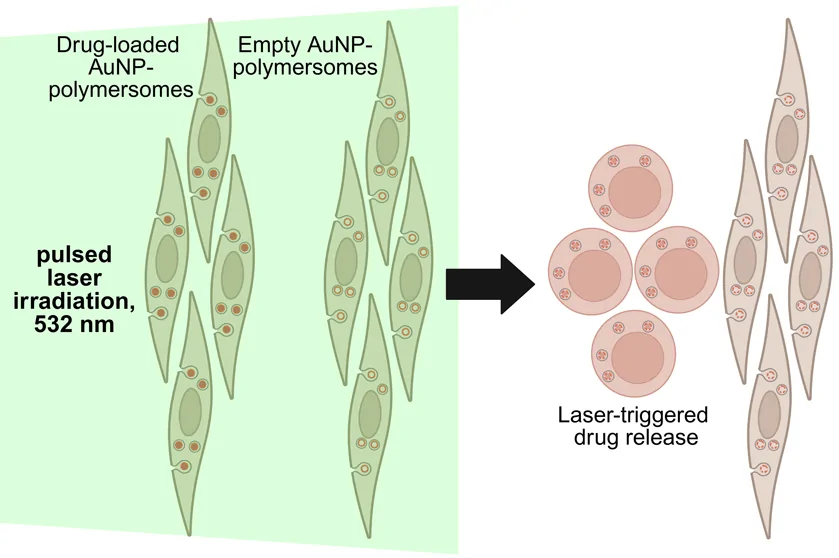

## Introduction

Molecular cargo delivery systems aim to enhance therapeutic efficacy by increasing local drug concentration at target sites while minimizing systemic side effects.^1,2^ One method of accomplishing cargo delivery is with self-assembled nanostructures such as liposomes or polymersomes.^3^ Polymersomes are spherical vesicles self-assembled from synthetic amphiphilic block copolymers, that offer several advantageous properties over their closely related natural structures i.e., liposomes. Thick bilayer membranes, low membrane permeability, mechanical and chemical stability, and tunability in chemical composition, make polymersomes a favorable drug delivery system.^4–7^ Many therapeutic agents exhibit limited cellular internalization due to factors such as molecular size, charge, chemical structure, and interactions with the biological environment,^8^ motivating the development of carrier vesicles as intracellular delivery vehicles.

Polymersome bilayers contain two regions for potential encapsulation, the hydrophobic inner region of the membrane which allows for loading of hydrophobic constituents, and an aqueous lumen which facilitates the encapsulation of hydrophilic drugs.^5,9^ Due to the synthetic nature of these copolymers, the chemical composition and structure can be tailored for specific applications.^6^ As a result, polymersomes can be engineered to bring about cargo release via various modalities where specific environmental cues trigger structural changes and cargo release. Such stimulus-responsive systems have been developed to respond to a variety of stimuli,^10–12^ for example changes in pH,^7,13–16^ temperature,^17–21^ or the application of light.^22–24^ The ability to trigger the release of vesicular content with light has recently gained attention, as light can be biocompatible, tunable, and has the potential to be controlled with high spatiotemporal precision.^25,26^ Photo-triggered release strategies in vesicular and polymeric delivery systems include photoisomerization caused by incorporation of an azobenzene chromophore,^27^ photocleavage reactions,^28–30^ and photopolymerization.^31^ Additionally, light has proven to be an effective strategy in the field of theranostics.^32^

In the case of polymersomes, one such method to render the vesicle photosensitive is by the inclusion of metallic nanoparticles that can compromise membrane integrity upon photoexcitation.^33–35^ Noble metal nanoparticles, particularly gold, are useful photosensitizers due to their large absorption cross sections arising from the localized surface plasmon resonance (LSPR). Light absorption at wavelengths congruent with the LSPR can generate photothermal and photomechanical effects capable of inducing changes in nearby structures via direct thermal changes, mechanical disruption from nanoparticle fragmentation, or through secondary thermal effects such as cavitation bubble formation.^36,37^ These thermomechanical effects can be enhanced under pulsed irradiation, such as that utilized in this study. In contrast to continuous wave irradiation commonly employed as a photo-stimulus, pulsed irradiation enables rapid energy deposition on time scales shorter than characteristic thermal diffusion times, thereby promoting localized membrane destabilization while minimizing heating of the surrounding environment. Recently, Uzel et al. demonstrated single-pulse nanosecond laser-stimulated release of doxorubicin from hybrid lipid nanoparticles containing 5 nm AuNPs, highlighting the potential of plasmonically sensitized vesicles for externally triggered intracellular drug delivery under pulsed irradiation conditions.^26^ Additionally, the strong near-field confinement and localized energy deposition associated with plasmonic nanostructures have enabled a variety of light-mediated nanoscale manipulation and delivery applications.^38^ One benefit of using gold nanoparticles (AuNPs) as photosensitizers is that the wavelength response can be tuned through the size, shape, and organization of the incorporated nanostructures, enabling photo-responsive systems spanning visible to near-infrared wavelengths. For example, gold nanorods can be utilized in drug delivery systems to enable NIR-triggered activation and cargo release.^39^

In the case of vesicles comprised of bilayer membranes, AuNPs can be loaded into the hydrophobic region of the membrane, for example, through the incorporation of dodecanethiol-functionalized spherical AuNPs.^35,40,41^ Dodecanethiol-functionalized spherical gold nanoparticles loaded into the membrane of micron-sized polymersomes have previously been demonstrated to render the vesicles responsive to pulsed laser irradiation.^33^ Two regimes of membrane disruption capable of inducing cargo release were observed: (1) complete vesicle rupture, and (2) the formation of transient pores that allow for release while the vesicle retains its spherical morphology.^33^ The response of these systems is strongly dependent on irradiation conditions, particularly the time scale of energy deposition.

A recent study from our group investigated the effects of picosecond pulsed laser irradiation on nano-polymersomes containing hydrophobic gold nanoparticles (AuNPs) in the membrane, a system closely related to the one examined in this study.^42^ The previous work by Salzer et al. demonstrated that membrane disruption could be controlled through both pulse energy and the extent of gold nanoparticle incorporation within the membrane. Higher per-pulse energies and increased AuNP concentrations generally resulted in a greater likelihood of complete membrane disruption, as demonstrated by dynamic light scattering and cryogenic transmission electron microscopy. These findings provide a useful framework for extending this irradiation-responsive AuNP-nanovesicle platform into biological systems.^42^

In this work, nanoscale polymersomes self-assembled from poly­(butadiene)_33_-*b*-poly­(ethylene oxide)_20_ were loaded with 3–5 nm dodecanethiol-functionalized spherical gold nanoparticles in the membrane and the chemotherapy drug doxorubicin in the aqueous core (doxorubicin-loaded AuNP-polymersomes). This particular diblock copolymer was selected because it forms stable polymersomes with a hydrophilic PEO corona that supports biocompatibility and prolonged circulation while providing an aqueous core suitable for encapsulation of hydrophilic therapeutics such as doxorubicin.^6^ Additionally, vesicles self-assembled from this diblock copolymer are minimally responsive to various stimuli, allowing controlled responses to light through the incorporation of AuNP photosensitizers. AuNP-polymersomes were shown to be effectively internalized by NIH 3T3 cells with minimal toxicity. Upon irradiation at a wavelength coinciding with the LSPR of the incorporated AuNPs, cell viability was reduced in cells treated with doxorubicin-loaded AuNP-polymersomes compared with doxorubicin-free AuNP-polymersome controls, consistent with irradiation-triggered doxorubicin release. This study demonstrates the utility of pulsed laser irradiation for controlled drug release in a cellular model and establishes pulsed plasmonic activation as a promising approach for externally-triggered intracellular cargo delivery.

## Results and Discussion

### Polymersome Characterization

Prior to intracellular release experiments, AuNP-polymersomes were self-assembled via a solvent injection self-assembly method and extensively characterized. Following self-assembly, the vesicles were dialyzed into PBS and analyzed via dynamic light scattering (DLS). DLS yielded a mean hydrodynamic diameter of 150 ± 2.76 nm with a polydispersity index of 0.148 ± .010 (Figure 1), indicating a monodisperse population of vesicles. Cryogenic transmission electron microscopy (cryo-TEM) was utilized to visualize the spherical vesicle morphology and confirm AuNP loading (Figure S1). The average vesicle diameter determined by cryo-TEM image analysis was previously reported to be approximately 50 nm with a membrane thickness of 11.39 nm.^42^ UV-Vis spectroscopy was utilized to determine the wavelength of the LSPR of the loaded AuNPs which showed a distinct peak at 544 nm (Figure S2). This absorption profile confirms that irradiation at 532 nm occurs near the LSPR of the AuNPs, providing efficient plasmonic excitation. To determine colloidal stability and whether doxorubicin loading altered the surface properties of the polymersomes, the zeta potentials of dialyzed doxorubicin-free AuNP-loaded polymersomes and doxorubicin-loaded AuNP-polymersomes were measured. Doxorubicin-free AuNP-loaded polymersomes exhibited a zeta potential of −5.60 ± 1.95 mV, while doxorubicin-loaded AuNP-polymersomes exhibited a comparable zeta potential of −5.01 ± 0.94 mV, indicating that encapsulation of doxorubicin did not substantially alter the surface properties of the vesicles. These zeta potentials are comparable to previous measurements of PBd-*b*-PEO polymersomes, where zeta potentials near −5 mV were reported and colloidal stability was attributed primarily to steric stabilization provided by the hydrated PEO corona rather than electrostatic repulsion.^43^

**Figure 1:**
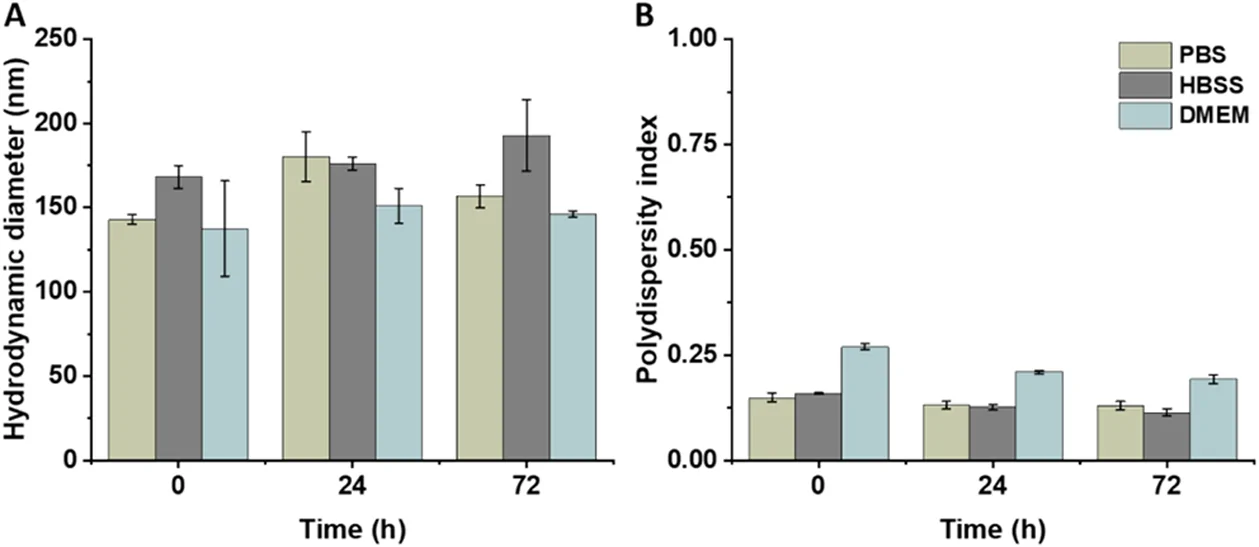
Stability of polymersomes in common cell culture buffers, including phosphate-buffered saline (PBS), Hank’s balanced salt solution (HBSS), and Dulbecco’s Modified Eagle Medium (DMEM). Dynamic light scattering (DLS) measurements were collected after 0, 24, and 72 h and analyzed for (A) hydrodynamic diameter (number-weighted) and (B) polydispersity index (PDI). All samples were prepared as described in the methods section and stored at 4 °C between measurements. Error bars represent the standard deviation of *N* = 3 independent samples. Minimal changes in size or polydispersity were observed across all buffer conditions.

As exposure to cell culture media is unavoidable for intracellular delivery applications, polymersome stability was evaluated in common cell culture buffers over 72 h using hydrodynamic size and polydispersity index as indicators of structural integrity. Polymersomes diluted in PBS remained stable over the maximum measured time interval of 72 h, exhibiting a minimally increased mean hydrodynamic diameter of 159 nm and a PDI of 0.136 (Figure 1 A,B). Similarly, polymersomes diluted in HBSS showed comparable trends in size and PDI, indicating minimal disruption to vesicle structure under these conditions (Figure 1 A,B). Polymersomes diluted in DMEM supplemented with FBS exhibited minimal size changes with an increased PDI of 0.224 (Figure 1 A,B). However, this increase in polydispersity is likely attributable to the presence of serum proteins and other dissolved media components; to confirm this, DLS was performed on control DMEM/FBS samples which showed a peak around 5.2 nm with a PDI of 0.443. Because DLS measurements are fundamentally intensity-weighted and cannot fully resolve multimodal distributions, the presence of smaller serum-associated species likely broadened the measured distribution. Despite the increased PDI observed in DMEM, the overall size range remained consistent across all tested conditions, suggesting that dilution into biologically relevant media does not significantly compromise polymersome stability, consistent with known structural stability.^5,7,16,34^ This stability is critical for downstream biological applications where exposure to serum-containing environments is unavoidable.

### Encapsulation Efficiency and Drug Loading Percent

A doxorubicin standard curve was generated over the concentration range of 0.225–0.523 mg/mL (Figure S3). Encapsulation efficiency was determined using an indirect subtraction method in which the mass of unencapsulated doxorubicin recovered following centrifugal ultrafiltration was quantified by UV-Vis spectroscopy and compared with the initial doxorubicin mass (Figure S4). The encapsulation efficiency was calculated to be 32.4% ± 2.7%, which is comparable to values reported for other doxorubicin-loaded polymersome formulations.^14,44^ Using the calculated encapsulated doxorubicin mass, the drug loading percentage was determined relative to the total carrier mass (0.30 mg PBd-*b*-PEO, 0.35 mg AuNPs, and the calculated mass of encapsulated doxorubicin), yielding a value of 14.2% ± 1.0%.

### Polymersome Internalization into NIH 3T3 Cells

To assess cellular internalization, NIH 3T3 cells were incubated with fluorescent Nile Red-labeled AuNP-polymersomes and imaged using confocal laser scanning microscopy followed by a z-stack reconstruction. NucBlue was used to stain the cell nuclei, while Nile Red fluorophore was loaded into the polymersome membrane for purposes of identifying the polymersomes (Figure 2). Overlay images demonstrated cellular localization of Nile Red fluorescence relative to the nuclear stain, indicating association of the polymersomes with the cells. Z-stack cross-sectional analysis further revealed a three-dimensional distribution of polymersome-associated fluorescence surrounding the nucleus, consistent with intracellular localization rather than exclusively surface-associated fluorescence (Figure 2). The observed perinuclear localization is consistent with size-dependent endocytic uptake mechanisms reported for nanoscale particles.^45^ To evaluate polymersome uptake kinetics, cells were incubated with polymersomes for varying durations, after which extracellular polymersomes were removed by washing and the cells were fixed. Fluorescence microscopy images were acquired at each time point and analyzed to determine the mean fluorescence intensity on a per-cell basis. The resulting uptake profile was plotted as a function of incubation time (Figure S5) and fit using a one-phase association model.^46^ Fluorescence intensity approached a plateau after approximately 15 min, indicating rapid cellular uptake. The fitted rate constant (*k*) was determined to be 0.25 ± 0.11 min^–1^, corresponding to a calculated uptake half-time (*t*_1/2_) of 2.8 min. The rapid uptake kinetics indicate that incubation periods exceeding 15 min result in maximal intracellular polymersome accumulation.

**Figure 2:**
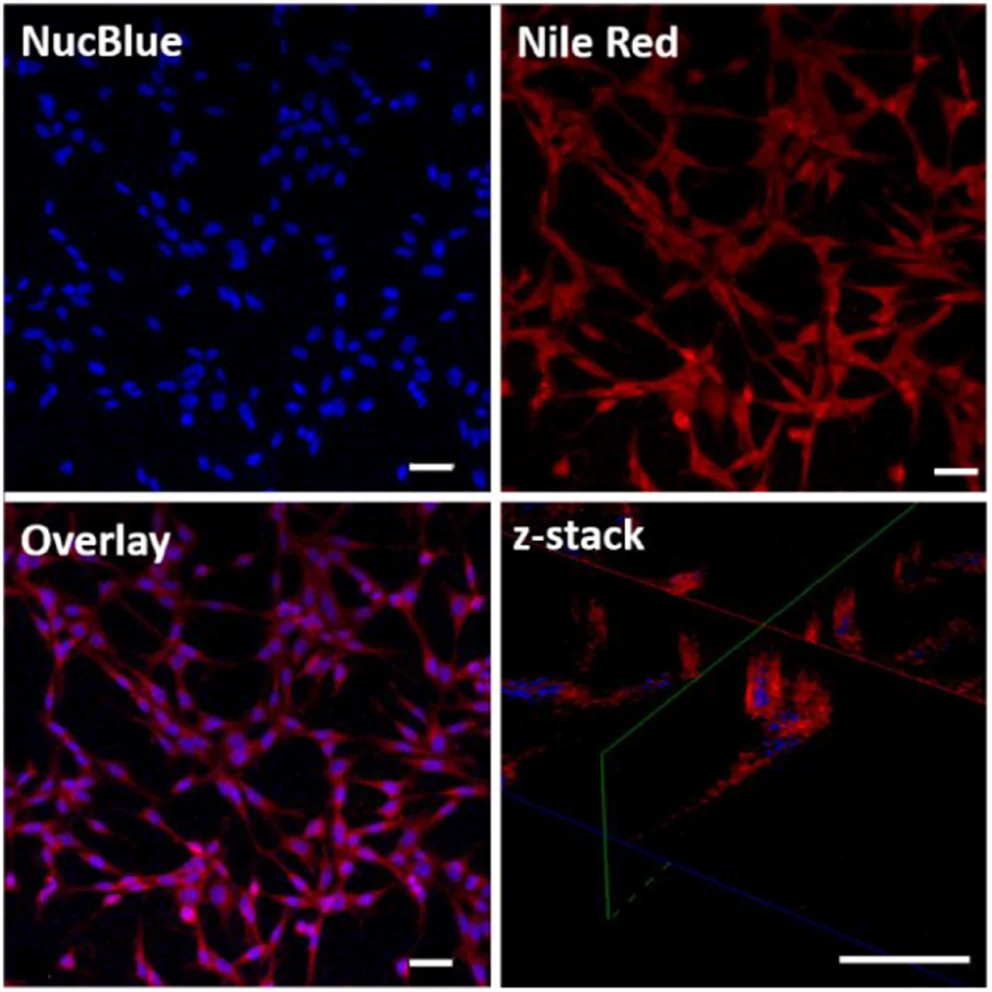
Confocal laser scanning microscopy images of NIH 3T3 cells following incubation with Nile Red-labeled polymersomes to assess cellular uptake. Cells were incubated for 1 h with Nile Red-labeled polymersomes (red) and stained with 0.5× NucBlue (blue). Representative images showing the NucBlue channel (top left), Nile Red channel (top right), and merged overlay (bottom left). Three-dimensional z-stack reconstruction (bottom right) illustrates the spatial distribution of the Nile Red signal relative to the nucleus. Scale bar: 50 μm.

### Laser Irradiation Setup and Controls

To achieve reproducible and sterile irradiation conditions, the cells were irradiated from the bottom of the plate upward while keeping the top of the plate covered. A 1000 mm focusing lens was used to generate a quasi-collimated beam at the bottom of the well with a beam size much smaller than the diameter of the well to achieve higher energy densities when required. To ensure uniform irradiation across the well surface, a galvanometer system was used to raster the beam across the well while maintaining an approximate 50% beam overlap between pulses (Figure 3 A).

**Figure 3:**
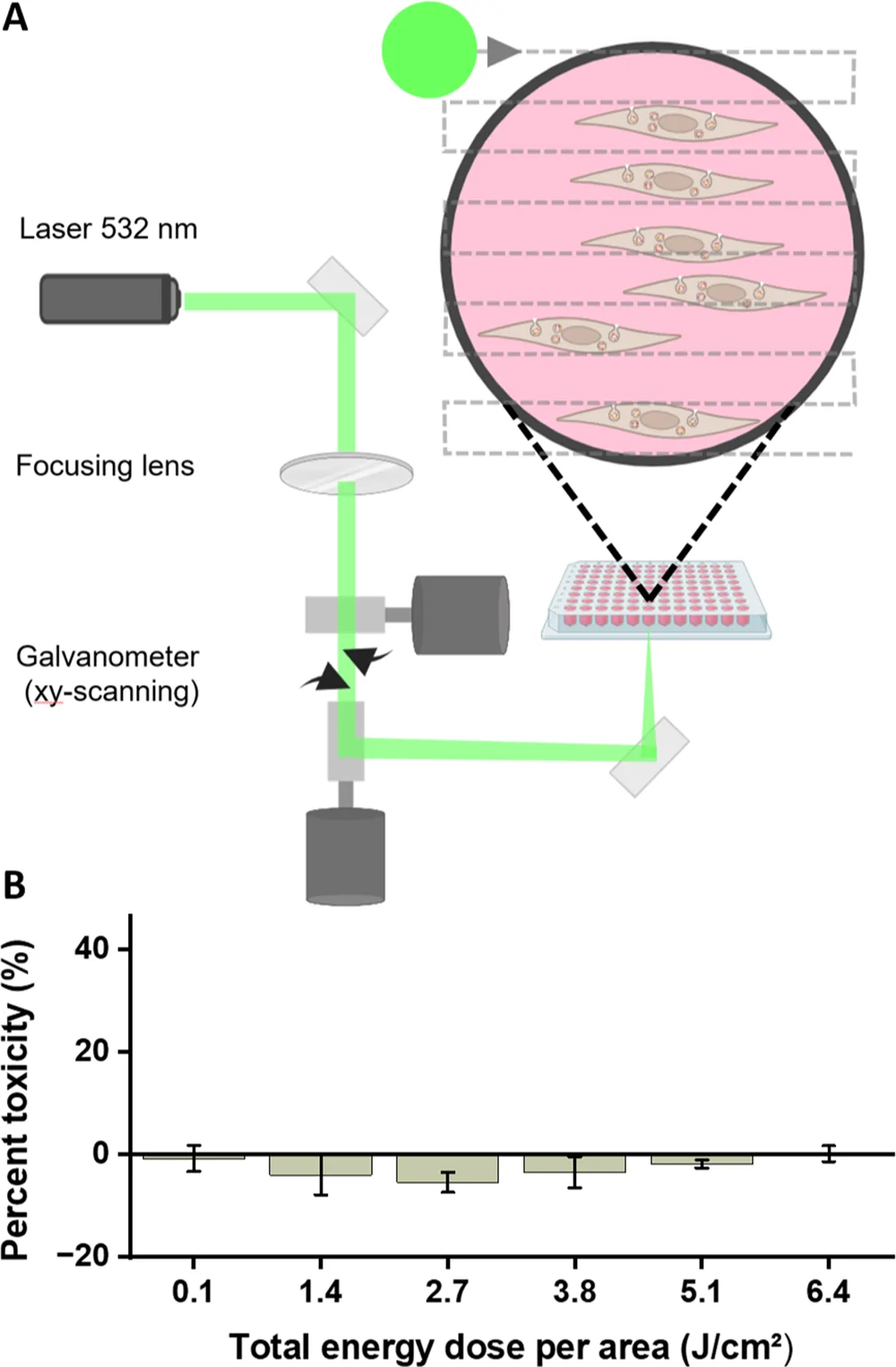
Laser irradiation set up and cellular response to irradiation. (A) A DAQ-controlled galvanometer moves two mirrors to allow for x–y rastering and uniform well irradiation. (B) NIH 3T3 cells were irradiated at the indicated total energy doses and incubated for 24 h prior to LDH analysis. Absorbance values were baseline-corrected by subtracting media controls and normalized to untreated cells. Error bars represent the standard deviation of three independent experiments (*N* = 3). Minimal LDH release was observed across all conditions, indicating low cytotoxicity associated with laser irradiation.

In order to assess the baseline toxicity of picosecond irradiation, NIH 3T3 cells were exposed to total doses up to 6.4 J/cm^2^ followed by 24 h incubation prior to toxicity analysis determined by LDH assay. Cytotoxicity remained low across all irradiation conditions, indicating minimal laser-induced toxicity under the tested parameters (Figure 3 B). Similarly, nanosecond irradiations indicated a potential dose window below 18 J/cm^2^ where no laser toxicity was observed.

To further evaluate passive leakage and baseline toxicity, cells were incubated with either doxorubicin-loaded or doxorubicin-free AuNP-polymersomes for 1 h and subsequently cultured for 24 h prior to measuring viability. In unirradiated samples, neither condition resulted in a measurable decrease in viability relative to untreated controls (Figure 4 A,B), indicating minimal passive release of doxorubicin in the absence of irradiation. Together, these findings demonstrate that both the AuNP-polymersome system and the applied picosecond irradiation conditions are independently well tolerated, establishing a baseline in which observed cytotoxic effects are consistent with triggered cargo release rather than intrinsic toxicity of the carrier system or laser exposure. Additionally, the AuNP-polymersome control samples exhibited minimal reductions in cell viability under both nanosecond and picosecond irradiation conditions, indicating that the combination of laser irradiation, embedded AuNPs, and polymersome exposure is minimally toxic (Figure 4 A,B).

**Figure 4:**
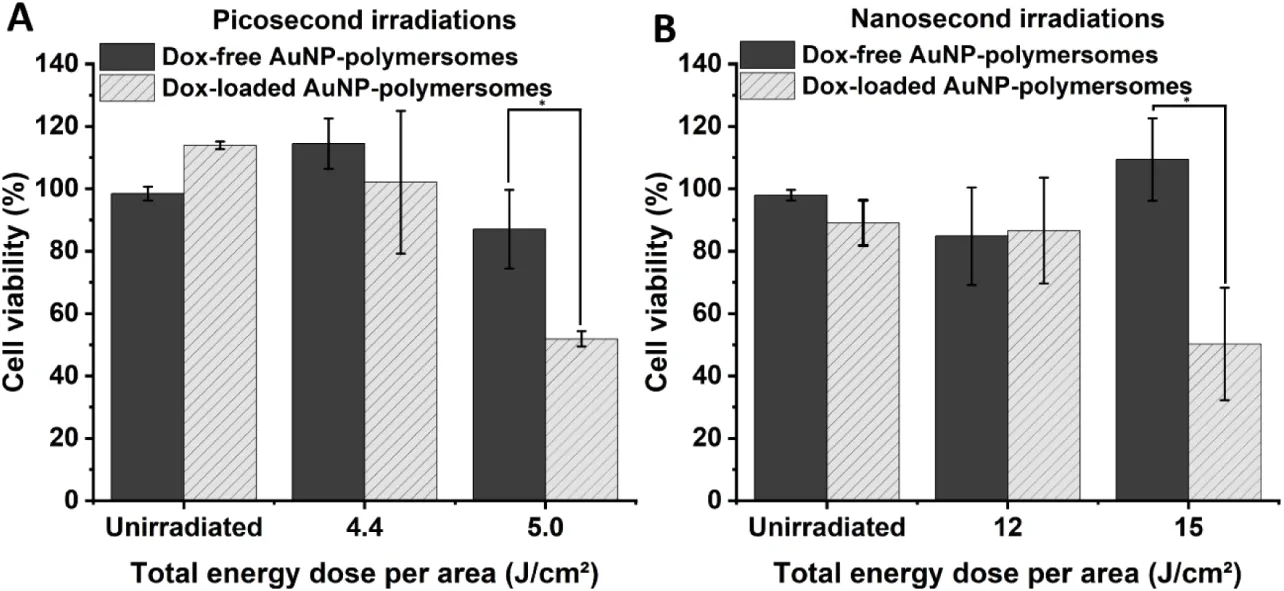
Cell viability following irradiation-induced cargo release from doxorubicin-loaded AuNP-polymersomes. Cell viability was assessed by MTT assay 24 h following irradiation and normalized to unirradiated control cells (N = 3) for (A) picosecond irradiation and (B) nanosecond irradiation. Under both laser conditions, minimal changes in viability were observed for irradiated control groups and doxorubicin-free AuNP-polymersomes (featuring AuNPs loaded into the polymersome membrane), whereas significant reductions in viability were observed for irradiated doxorubicin-loaded AuNP-polymersomes. These results are consistent with irradiation-triggered intracellular release of doxorubicin at total irradiation doses of 5.0 J/cm^2^ (picosecond) and 15 J/cm^2^ (nanosecond). Error bars represent the standard deviation of three independent experiments. * denotes P < 0.05.

### Evaluation of Irradiation-Triggered Intracellular Doxorubicin Release

To evaluate light-triggered intracellular cargo release, polymersomes were loaded with doxorubicin and incubated with NIH 3T3 cells prior to picosecond or nanosecond laser irradiation. Following irradiation, cells were incubated for 24 h before assessing morphology by phase contrast microscopy and quantifying viability using an MTT assay. Doxorubicin-free AuNP-polymersomes were included as an internal control to distinguish cytotoxicity associated with doxorubicin release as opposed to cytotoxicity that resulted from the combined effects of AuNP-polymersome exposure and laser irradiation. An AuNP-only control was not feasible because the dodecanethiol-functionalized AuNPs used in this system are hydrophobic and cannot be stably dispersed in aqueous cell culture media.

At a total dose of 4.4 J/cm^2^, only minor differences in viability were observed between AuNP-polymersomes with or without doxorubicin loading, suggesting limited release or biological response under these picosecond laser conditions (Figure 4 A). At lower total doses, no changes were observed in either population of cells. Increasing the total energy dose per area to 5.0 J/cm^2^ resulted in pronounced morphological changes only in cells treated with doxorubicin-loaded AuNP-polymersomes, as shown by increased cell rounding and detachment, consistent with reduced cellular health (Figure 5 A,B). This observation was corroborated by MTT analysis, which demonstrated a reduction in viability to approximately 50% for doxorubicin-loaded AuNP-polymersomes (Figure 4 A, p = 0.047). Given the minimal toxicity observed from doxorubicin-free AuNP-polymersomes and laser irradiation independently, this reduction in viability is consistent with picosecond irradiation-triggered intracellular release of doxorubicin from the AuNP-loaded nano-polymersomes. Increasing the total energy dose to 5.6 J/cm^2^ resulted in nonspecific cytotoxicity from cells which had internalized doxorubicin-free AuNP-polymersomes. NIH 3T3 cells, due to their slower rates of proliferation than cancer cell lines, typically have a lower susceptibility to doxorubicin. They have been shown to exhibit a sigmoidal-like dose dependence, with an initial shallow decline in viability, a steeper transition region at intermediate doxorubicin concentrations, and a more gradual decrease at higher concentrations.^47^ This behavior, combined with the threshold-like nature of rupture in this nano-polymersome carrier system,^42^ may explain the pronounced difference in cell viability observed between picosecond laser energy doses of 4.4 and 5.0 J/cm^2^.

**Figure 5:**
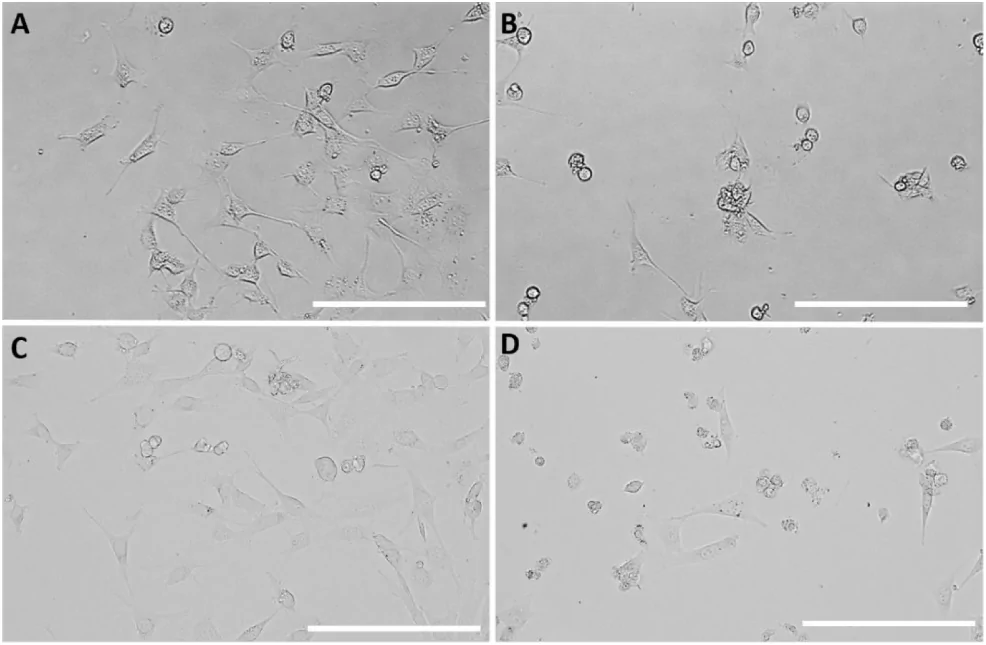
Representative phase contrast images of NIH 3T3 cells incubated with either doxorubicin-free AuNP-polymersomes or dox-loaded AuNP-polymersomes. (A) Representative image of NIH 3T3 cells incubated with doxorubicin-free AuNP-polymersomes and irradiated with a picosecond laser at 5.0 J/cm^2^. (B) Representative image of NIH 3T3 cells incubated with dox-loaded AuNP-polymersomes and irradiated with a picosecond laser at 5.0 J/cm^2^. (C) Representative image of NIH 3T3 cells incubated with doxorubicin-free AuNP-polymersomes and irradiated with a nanosecond laser at 15 J/cm^2^. (D) Representative image of NIH 3T3 cells incubated with dox-loaded AuNP-polymersomes irradiated with a nanosecond laser at 15 J/cm^2^. Scale bar: 200 μm. Cell morphology representing reduced viability was identified in doxorubicin samples post irradiation.

To determine whether cargo release could be achieved with longer pulse duration irradiations, analogous experiments were performed using nanosecond laser irradiation. As with the picosecond studies, NIH 3T3 cells were incubated with either doxorubicin-free AuNP-polymersomes or doxorubicin-loaded AuNP-polymersomes prior to irradiation and evaluated by phase contrast microscopy with viability confirmed via an MTT assay. At total doses below 12 J/cm^2^, minimal reductions in viability were observed in either group (Figure 4 B). At doses of 18 J/cm^2^ and above, substantial nonspecific cytotoxicity became evident irrespective of cargo loading, which may be partially attributed to observed plate breakdown by the laser.

At total doses of 15 J/cm^2^, phase contrast microscopy revealed substantial morphological changes in cells treated with doxorubicin-loaded AuNP-polymersomes (Figure 5 C,D). These observations were supported by MTT analysis, which demonstrated a reduction in viability to approximately 50% (Figure 4 B, p = 0.008). Given the minimal toxicity observed for doxorubicin-free AuNP-polymersomes at this dose, the decrease in viability is consistent with intracellular release of doxorubicin following nanosecond irradiation.

Notably, the total irradiation dose required to observe selective cytotoxicity under nanosecond irradiation (15 J/cm^2^) was substantially greater than that required for picosecond irradiation (5.0 J/cm^2^), despite the higher per-pulse fluence associated with the nanosecond laser (150 vs 48 mJ/cm^2^). One possible explanation is the increased thermal confinement associated with picosecond irradiation.^36^ Under very short pulse durations, energy is delivered over a time scale which is comparable to the phonon–phonon coupling time (on the order of 100’s of ps). This limits thermal diffusion during the pulse and can produce a more rapid increase in nanoparticle temperature, potentially resulting in an abrupt thermomechanical response which can bring about rapid boiling or phase explosion. In contrast, during nanosecond irradiation, greater thermal diffusion occurs during the pulse, allowing a larger fraction of the absorbed energy to dissipate into the surrounding environment as heat and reducing the peak temperatures reached by the nanoparticle. Consequently, picosecond irradiation will produce stronger thermomechanical stresses in the vicinity of the membrane-incorporated AuNPs bringing about destabilization of the polymer membrane. This can reduce the total irradiation dose required to achieve intracellular cargo release compared with longer pulse duration nanosecond irradiation. Although direct elucidation of the underlying release mechanism was beyond the scope of this study, these observations suggest that pulse duration influences the irradiation conditions necessary to achieve intracellular cargo release. Together, the picosecond and nanosecond results demonstrate light-triggered intracellular cargo release from doxorubicin-loaded AuNP-polymersomes across multiple pulsed laser regimes, with pulse duration influencing the irradiation threshold required to elicit a biological response.

In this study, nanosecond and picosecond pulse irradiations were evaluated as cargo release stimuli because they represent commonly employed pulsed laser regimes in biomedical applications.^48^ Nanosecond lasers have broad biomedical and clinical use, contributing to their widespread availability, and are utilized in plasmonic applications such as photoacoustic imaging, and nanoparticle-mediated photothermal therapies.^37,48^ Picosecond irradiation has similarly attracted interest for nanoparticle-mediated delivery and photomechanical therapies.^26,37^ Because these pulse durations span distinct irradiation regimes with different energy deposition characteristics,^36^ observing biological responses consistent with irradiation-triggered cargo release under both conditions broadens the potential applicability of the AuNP-polymersome platform. Collectively, these findings suggest that the system is compatible with multiple pulsed laser modalities relevant to translational biomedical applications.

## Conclusions

This work demonstrates a pulsed laser-responsive AuNP-polymersome platform that produces selective irradiation-dependent biological responses consistent with triggered intracellular cargo release. The AuNP-polymersomes exhibited favorable stability in common cell culture media, efficient cellular internalization, and minimal inherent cytotoxicity while enabling irradiation-triggered release of encapsulated doxorubicin. The incorporation of membrane-embedded AuNPs as plasmonic photosensitizers enables pulsed laser irradiation to trigger polymersome membrane destabilization and cargo release.

Future work will focus on systematically investigating the influence of laser parameters, including fluence and total irradiation dose, on membrane destabilization and cargo release behavior as well as evaluating cellular uptake and cargo delivery in additional cell lines. Because pulsed irradiation of AuNPs can access multiple energy dissipation pathways, including localized photothermal effects, nanoparticle fragmentation, and cavitation-related processes, tuning irradiation conditions may enable increasingly precise and controllable delivery responses. Future studies will also focus on direct visualization of intracellular cargo release, providing spatially resolved evidence of cargo redistribution following laser irradiation. Collectively, these findings establish a foundation for the development of externally triggered, spatiotemporally controlled polymersome delivery systems for biological applications.

## Materials and Methods

### Polymersome Self-Assembly

Nano-polymersomes were self-assembled via a solvent injection method.^42^ The diblock copolymer poly­(butadiene)_33_-*b*-poly­(ethylene oxide)_20_ (PBd_33_-*b*-PEO_20_; Polymer Source P43223B1-BdEO, QC, Canada) was dissolved in tetrahydrofuran (THF) at a concentration of 2 mg/mL (300 μL total volume), constituting the organic phase. Characterization of the diblock copolymer can be found in Figure S6. The polymer solution was added dropwise using a 3 mL syringe fitted with an 18G blunt-tip needle into 700 μL of 1× phosphate-buffered saline, the aqueous phase (PBS; Thermo Fisher Scientific, Waltham, MA) under constant magnetic stirring (350 rpm).

Hydrophobic and hydrophilic components can be incorporated during the self-assembly process. For example, dodecanethiol-functionalized gold nanoparticles (AuNPs, 2% w/v in toluene; Sigma-Aldrich, St. Louis, MO) were dried under nitrogen to remove the solvent and subsequently reconstituted in the THF polymer solution. Nile Red (5 μL of 1 mg/mL in THF; Thermo Fisher Scientific) was added to the organic phase to enable visualization of the hydrophobic bilayer region. Doxorubicin-loaded polymersomes were prepared by adding doxorubicin (1.79 mM before self-assembly, 1.25 mM final concentration) to the aqueous phase prior to assembly. Figure 6 depicts a schematic representation of the self-assembly, loading, and purification processes.

**Figure 6:**
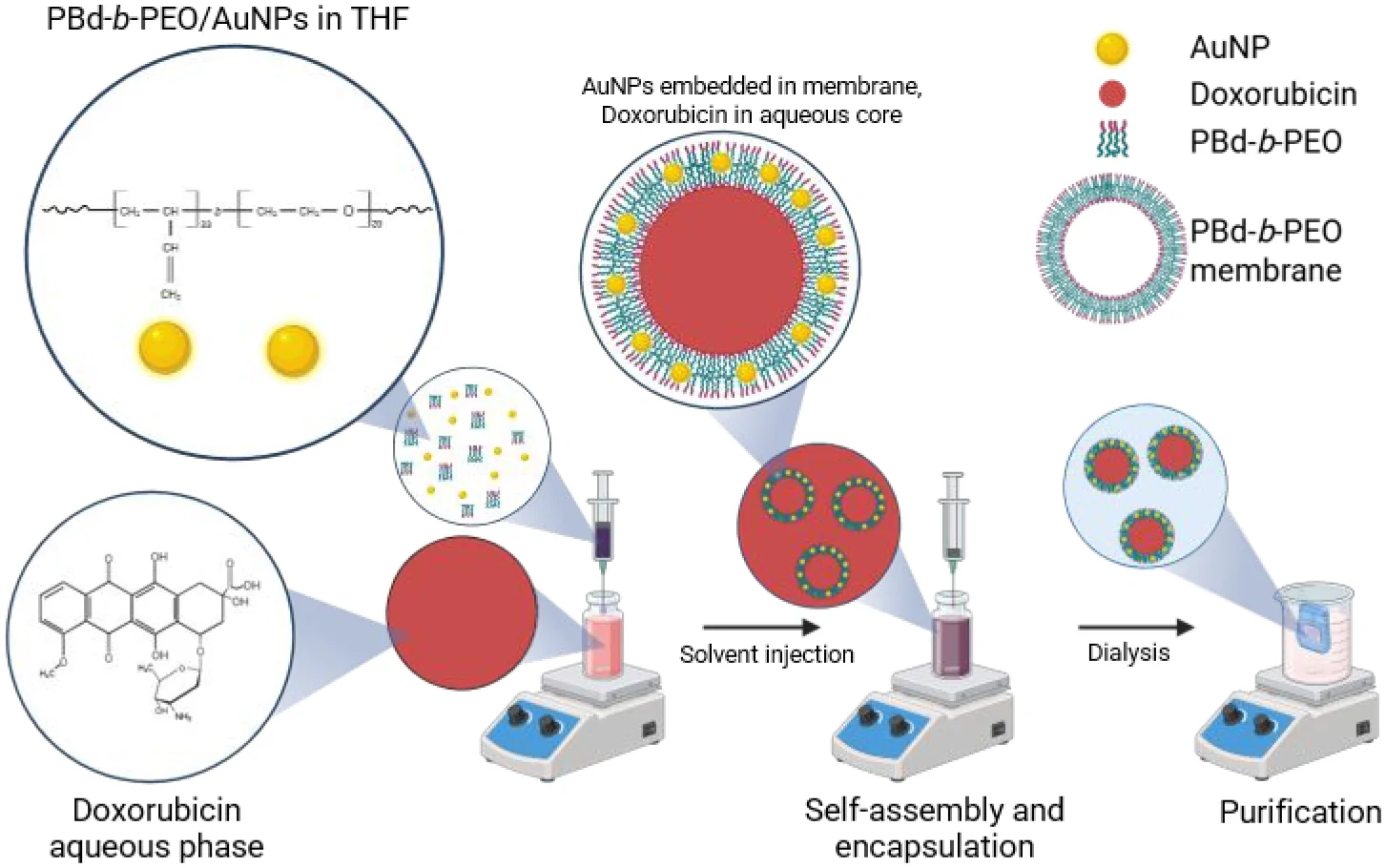
Schematic illustration of the solvent injection preparation of AuNP-loaded PBd-*b*-PEO polymersomes, encapsulation of doxorubicin during self-assembly, and removal of unencapsulated doxorubicin by dialysis.

### Polymersome Characterization

Following self-assembly, vesicle size and polydispersity were characterized using dynamic light scattering (DLS) on a Malvern Zetasizer Nano ZS at a scattering angle of 173°. Samples were diluted 33× in 1× PBS to a final volume of 1 mL in a polystyrene cuvette (Sigma-Aldrich). Measurements were performed in triplicate at 25 °C with 10 scans per run.

Successful assembly was defined by the presence of a prominent peak in the number-weighted size distribution in the range of 50–200 nm and a polydispersity index (PDI) below 0.250.

### Polymersome Buffer Exchange

To remove residual THF following self-assembly, polymersome suspensions were dialyzed using 20,000 MWCO Slide-A-Lyzer G2 dialysis cassettes (Thermo Fisher Scientific). Samples were dialyzed against 2 L of 1× PBS for 24 h at 4 °C with gentle stirring (Figure 6). In the case of doxorubicin-loaded polymersomes, an additional dialysis step against 2 L of water was added before dialysis with PBS. After dialysis, samples were re-characterized by DLS to confirm structural stability.

### Polymersome Stability

Polymersome stability was evaluated in cell culture media by diluting samples 33× into: (i) 1× PBS, (ii) Hanks’ Balanced Salt Solution (HBSS; VWR, Radnor, PA), or (iii) Dulbecco’s Modified Eagle Medium (DMEM; Sigma-Aldrich) supplemented with 10% fetal bovine serum (FBS). Samples were monitored over 72 h using DLS to assess changes in size distribution and PDI.

The zeta potential of dialyzed doxorubicin-loaded AuNP-polymersomes and doxorubicin-free AuNP-polymersomes was measured using a Malvern Zetasizer. Samples were diluted 33-fold in 1× PBS and analyzed using a dip cell (Malvern Zetasizer Nano Series Dip Cell, Universal ‘Dip’ Cell Kit, ZEN1002) according to the manufacturer’s recommendations. Measurements were performed at 25 °C using three independent measurements for each sample, and the reported values represent the mean ± standard deviation.

### Cryo-TEM

Prior to cryogenic transmission electron microscopy (cryo-TEM), AuNP-polymersome samples were concentrated 15-fold using a vacuum concentrator (Vacufuge Plus, Eppendorf). For vitrification, 5 μL of the concentrated sample was applied to a 400-mesh lacey carbon-coated copper TEM grid (Ted Pella, Redding, CA) and vitrified using a manual plunge freezer (EMS-001, Electron Microscopy Sciences, Hatfield, PA). Excess sample was removed by manual front-side blotting with filter paper before the grid was immediately plunged into liquid ethane maintained at −180 °C. Vitrified grids were transferred to a Gatan ELSA single-tilt cryo-holder and imaged using a JEOL JEM-2100Plus transmission electron microscope equipped with a Rio 4k camera (Gatan, Pleasanton, CA). Images were acquired at an accelerating voltage of 200 kV and a nominal magnification of 30,000×.^42^

### Encapsulation Efficiency and Drug Loading Percentage

To determine doxorubicin encapsulation efficiency, a standard curve was generated by serial dilution of a 1.70 mM doxorubicin stock solution (Figure S3) followed by determination of absorbance by UV-Vis spectroscopy (Agilent Technologies, Cary 60 UV-Vis). A 1.84 mM (1.26 mM final concentration after self-assembly) doxorubicin stock solution was then diluted 1:2 (v/v, doxorubicin:water) and its absorbance measured by UV-Vis spectroscopy to determine the initial doxorubicin concentration prior to polymersome self-assembly. Following self-assembly, unencapsulated doxorubicin was separated from the polymersomes by centrifugal ultrafiltration using 30 kDa molecular weight cutoff regenerated cellulose Amicon Ultra filters (MilliporeSigma). The filtrates were collected, dried, and resuspended to the original doxorubicin volume. These samples were subsequently diluted 1:2 (v/v, doxorubicin solution:water) and analyzed by UV-Vis spectroscopy (Figure S4). The mass of unencapsulated doxorubicin was determined from the standard curve and used to calculate the encapsulation efficiency according to eq 1.

1 EE(%)=mbefore−mfiltratembefore×100%

Where m_before_ is the initial mass of doxorubicin added during polymersome preparation and m_filtrate_ is the mass of unencapsulated doxorubicin recovered in the filtrate.

The mass of encapsulated doxorubicin was then used to calculate the drug loading percentage according to eq 2.

2 DL(%)=mdoxencap(mdoxencap+mpolymer+mAuNPs)×100%

where mpolymer and mAuNP are the masses of polymer and gold nanoparticles used during polymersome preparation, respectively while mdox_encap_ is calculated from the difference between m_before_ and m_filtrate_.

### Cell Culture and Polymersome Uptake

NIH 3T3 fibroblasts were cultured in DMEM with 10% FBS and seeded at a density of 50,000 cells/well into 24-well plates containing glass coverslips. Cells were incubated for 24 h prior to treatment.

For uptake studies, cells were incubated with the self-assembled polymersomes (1:5 dilution in DMEM) for 1 h, washed with PBS, and stained with NucBlue nuclear stain (Thermo Fisher Scientific). Coverslips were removed from the wells and mounted to secondary coverslips using antifade mounting medium (Biotium, Fremont, CA) and sealed prior to imaging.

### Confocal Microscopy

Cells were imaged using a confocal laser scanning microscope (Leica TCS system) equipped with a 20×/0.75 NA objective (HC PL APO CS2). Excitation was performed using a 405 nm Diode laser and a 561 nm DPSS laser. Emission was collected using HyD detectors with detection windows set to 408–516 nm and 635–700 nm. Z-stack imaging was performed using 13 slices with a step size of 1.04 μm. Three-dimensional reconstruction was conducted using LAS X software (version 3.5.7.23225) to confirm intracellular localization of polymersomes.

### Uptake Kinetics

NIH-3T3 cells were seeded onto glass coverslips (14,850 cells) placed in the wells of a 24-well plate and incubated overnight to allow for cell attachment. Nile Red-labeled polymersomes were added at the same concentration (1:11 v/v, polymersome:PBS) used in subsequent irradiation studies and incubated with the cells for the indicated time points. Following incubation, the polymersome suspension was removed, and the cells were washed twice with PBS to remove non-internalized polymersomes. Cells were then fixed with 3.7% formaldehyde for 10 min at room temperature. Following fixation, the cells were washed with PBS and incubated with 0.5 M ammonium chloride (NH_4_Cl) to quench residual aldehyde groups. After a final PBS wash, the coverslips were imaged using a fluorescence microscope (Nikon Eclipse Ti) under identical imaging settings for all samples.

Cell images were captured in both the phase-contrast and fluorescent channels. Phase-contrast images were analyzed using Cellpose-SAM^49^ to segment the image and create masks for individual cells. The masks were used to segment the fluorescent image, and the total fluorescence intensity of each cell was normalized to its area using a custom Matlab script (The Mathworks, Inc., version R2026a). A minimum of 15 cells were analyzed in each of three biological replicates. The uptake rate constant was determined by fitting the time-dependent change in fluorescence to a one-phase association model:

3 $$F\left(t\right)=F_{0}+\left(F_{max}-F_{0}\right)\left(1-e^{-kt}\right)$$

where F is fluorescence, F_0_ is the background fluorescence, *F*_max_ is the maximal fluorescence, and k is the rate constant (OriginPro, OriginLab, version 2024b).

### Cytotoxicity Assay

Cytotoxicity was evaluated using a lactate dehydrogenase (LDH) assay (Thermo Fisher Scientific) following the manufacturer’s protocol. Cells (approximately 1500) were seeded in 96-well plates and incubated for 24 h prior to treatment.

Each absorbance term in eq 4 corresponds to a measured value at 490 nm with background correction at 680 nm, i.e., Abs_490_ – Abs_680_. Spontaneous LDH release was determined from untreated cells, while maximum LDH release was obtained from cells treated with the supplied lysis buffer.

Percent cytotoxicity was calculated per eq 4 as

4 $$Cytotoxicity\left(\%\right)=\left(Abs_{sample}-Abs_{spontaneous}\right)/\left(Abs_{max}-Abs_{spontaneous}\right)\times 100\%$$

Values were calculated on a per well basis, averaged across replicates (n = 3), and reported as mean ± standard deviation.

### Laser-Induced Cargo Release

NIH 3T3 cells (∼2500 cells per well) were seeded in 96-well plates and incubated with doxorubicin-loaded polymersomes (10 μL) for 1 h. Samples were washed twice with PBS and replenished with DMEM with 10% FBS prior to irradiation.

Irradiation was performed using either a picosecond or nanosecond pulsed laser operating at 532 nm. Picosecond irradiation was conducted using a frequency-doubled Nd:YAG laser (Ekspla PL2241, Vilnius, Lithuania) producing 25 ps pulses at a repetition rate of 250 Hz and pulse energies of 0.7 or 0.8 mJ. Nanosecond irradiation was performed using a frequency-doubled Nd:YAG laser (Ekspla NL303, Vilnius, Lithuania) producing 4 ns pulses at a repetition rate of 10 Hz and pulse energies of 35 or 45 mJ. The picosecond beam was passed through a 1000 mm plano-convex lens resulting in a beam diameter of 1.45 mm at the bottom window of the well. The beam diameter was determined using the knife edge method as previously published.^42^ For nanosecond irradiation, the beam was used without additional focusing optics and exhibited a beam diameter of 6.28 mm at the sample. To facilitate rastering of the beam across the well, the beam was directed through a dual-axis galvanometer mirror system (Thorlabs QS20XY-AG) controlled using a National Instruments data acquisition (DAQ) device.

Following irradiation, cells were incubated for 24 h prior to phase contrast imaging (Life Technologies, EVOS FL, Thermo Fisher) and performing MTT viability assessment.

### Laser Fluence and Dose Calculations

Pulse energies of 0.7–0.8 mJ (picosecond) and 35–45 mJ (nanosecond) correspond to per pulse fluences of approximately 42–48 and 110–150 mJ/cm^2^, respectively. Raster scanning was performed with 50% beam overlap, resulting in approximately four pulses incident on each location during a single pass with the software accounting for differences in laser repetition rate. The cumulative irradiation dose after 26 raster passes was calculated using eq 5:

5 $$D=N_{eff}\times F$$

where *D* is the cumulative delivered energy dose (J/cm^2^), *N*_*eff*_ is the effective number of pulses incident on a given area after accounting for pulse overlap and the number of raster passes, *F* is the single-pulse fluence (mJ/cm^2^). Using this approach, picosecond irradiation resulted in cumulative doses of approximately 4.4 and 5.0 J/cm^2^ for pulse energies of 0.7 and 0.8 mJ, respectively, while nanosecond irradiation resulted in cumulative doses of approximately 12 and 15 J/cm^2^ for pulse energies of 35 and 45 mJ, respectively.

### MTT Cell Viability Assay

Cell viability following irradiation was assessed using an MTT assay (Thermo Fisher Scientific). Media was removed and replaced with fresh DMEM with 10% FBS (100 μL), followed by addition of MTT stock solution (10 μL, 12 mM). Cells were incubated for 2 h at 37 °C.

Media was then removed, leaving approximately 25 μL in each well, and dimethyl sulfoxide (DMSO, 50 μL) was added to solubilize formazan crystals. Absorbance was measured at 540 nm using a CLARIOstar Plus plate reader.

Cell viability was calculated as per eq 6:

6 $$Cell\;viability\left(\%\right)=\left(Abs_{well}/Abs_{avg\;control}\right)\times 100\%$$

Values were calculated per well, averaged within biological replicates (n = 3), and reported as mean ± standard deviation. Statistical significance was assessed using a paired two-tailed Student’s t-test.

## Supplementary Material



## Notes

Cryo-TEM characterization of AuNP-loaded PBd-*b*-PEO polymersomes; UV-Vis spectrum of AuNP-loaded polymersomes; doxorubicin standard curve; UV-Vis analysis for encapsulation efficiency and drug loading; cellular uptake kinetics; ^1^H NMR and SEC characterization of PBd-*b*-PEO (PDF)
