# Supplementary material for: Plasmonic Nano-Polymersomes for Pulsed Laser-Triggered Intracellular Cargo Release
Source: ACS Omega. 2026 Sep 16;11(38):58171–80. doi: 10.1021/acsomega.6c08089 (PMC13629353; doi:10.1021/acsomega.6c08089)
Supplement: Supplementary file 1 [file ao6c08089_si_001.pdf]

Plasmonic Nano-Polymersomes for Pulsed Laser-Triggered Intracellular Cargo Release

Sebastian A. Zmijewski Jr.[1], Jesse Cecero[2], Sean M. O'Malley[1,2], Eric A. Klein[1,3], Julianne C. Griepenburg[1,2]*

[1]Rutgers University-Camden, Center for Computational and Integrative Biology, Camden, NJ 08102

[2]Rutgers University-Camden, Department of Physics, Camden, NJ 08102

[3]Rutgers University-Camden, Department of Biology, Camden, NJ 08102

*Corresponding author email: j.griepenburg@rutgers.edu

Supporting information

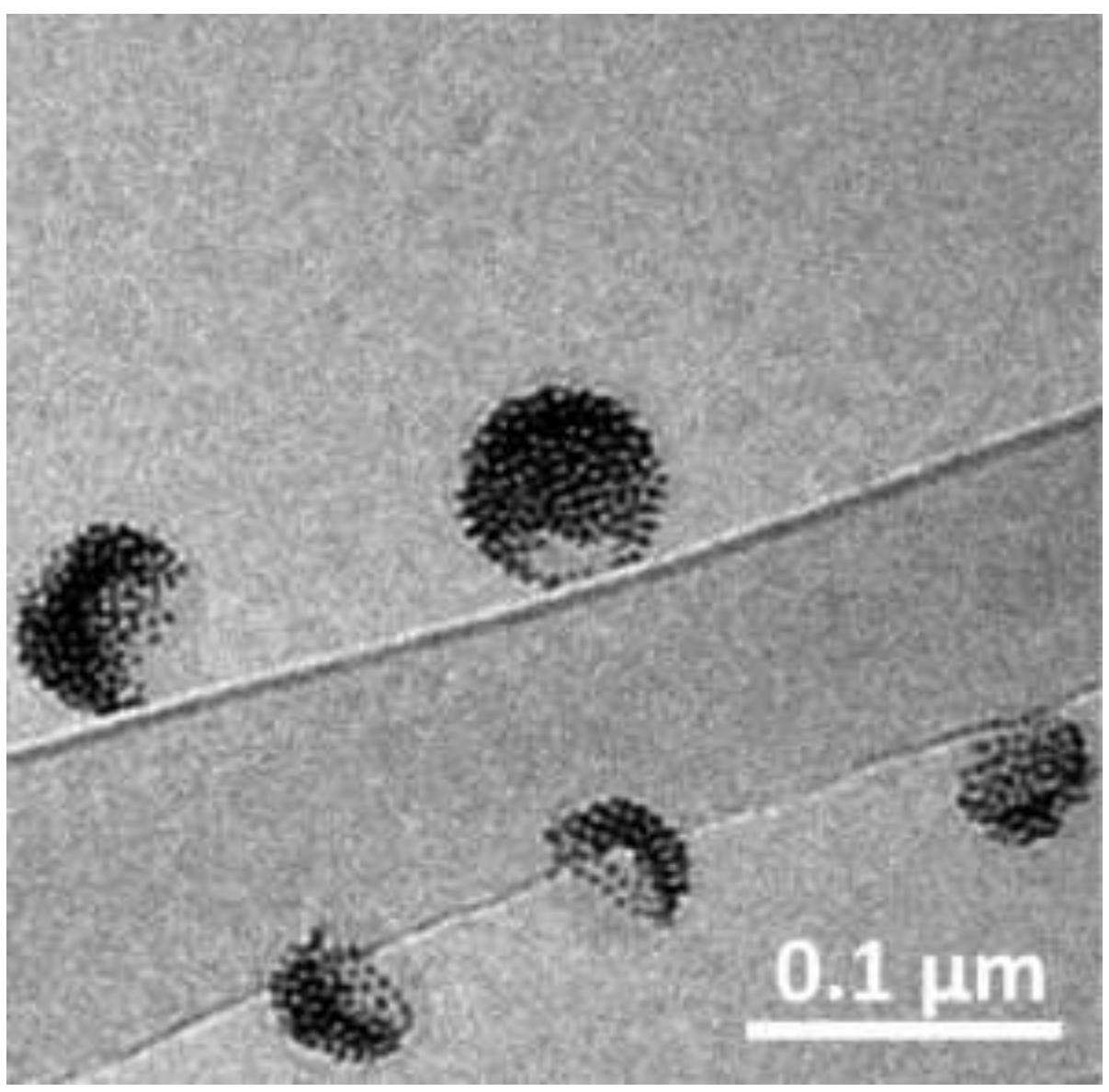

Figure S1. Representative cryogenic transmission electron microscopy (cryo-TEM) image of AuNP-loaded PBd-*b*-PEO polymersomes. Scale bar = 0.1 µm

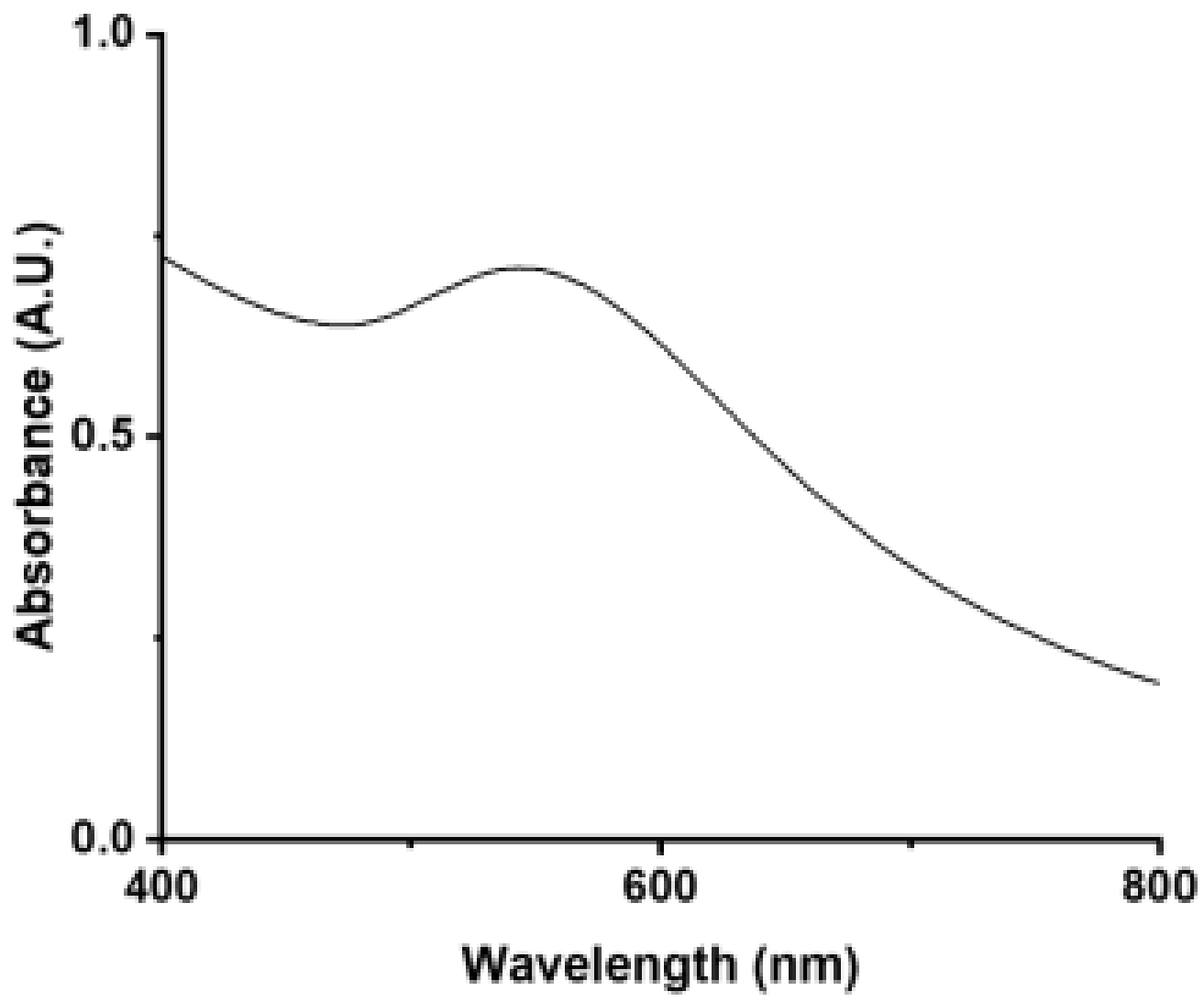

Figure S2. UV-Vis spectrum of AuNP-loaded polymersomes acquired using a short path length cuvette to minimize scattering from the vesicles. The LSPR peak of the membrane-embedded AuNPs was observed at 544 nm, showing strong spectral overlap with the 532 nm irradiation wavelength used in this study.

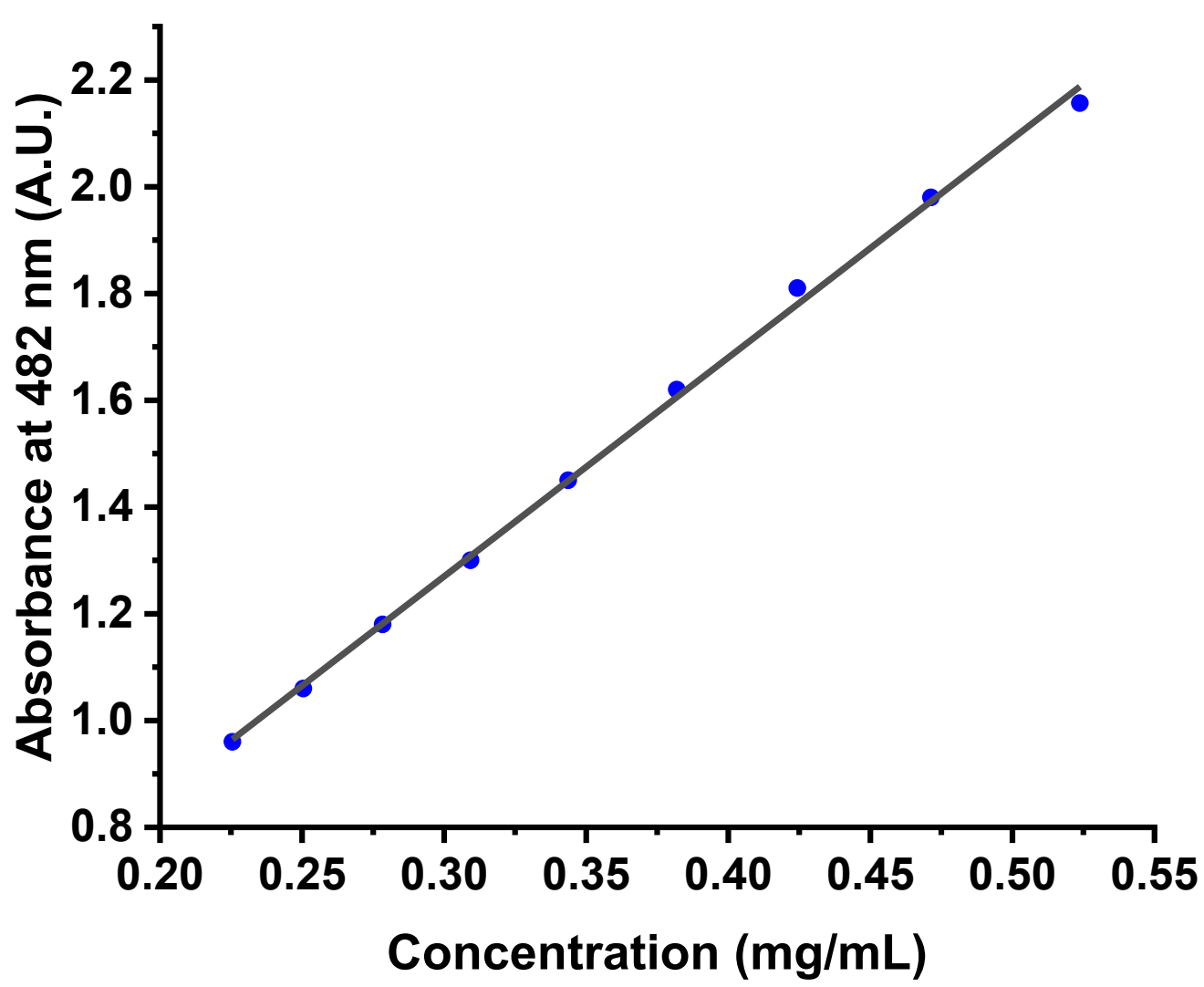

Figure S3. Absorbance standard curve of doxorubicin in water at 482 nm as a function of concentration. This standard curve was then used to determine the concentration of encapsulated doxorubicin. The equation for the line was determined to be y = 4.099x +.041 (R^2= 0.9984) where the slope represents the mass extinction coefficient, 4.10 mL mg^{-1} cm^{-1}.

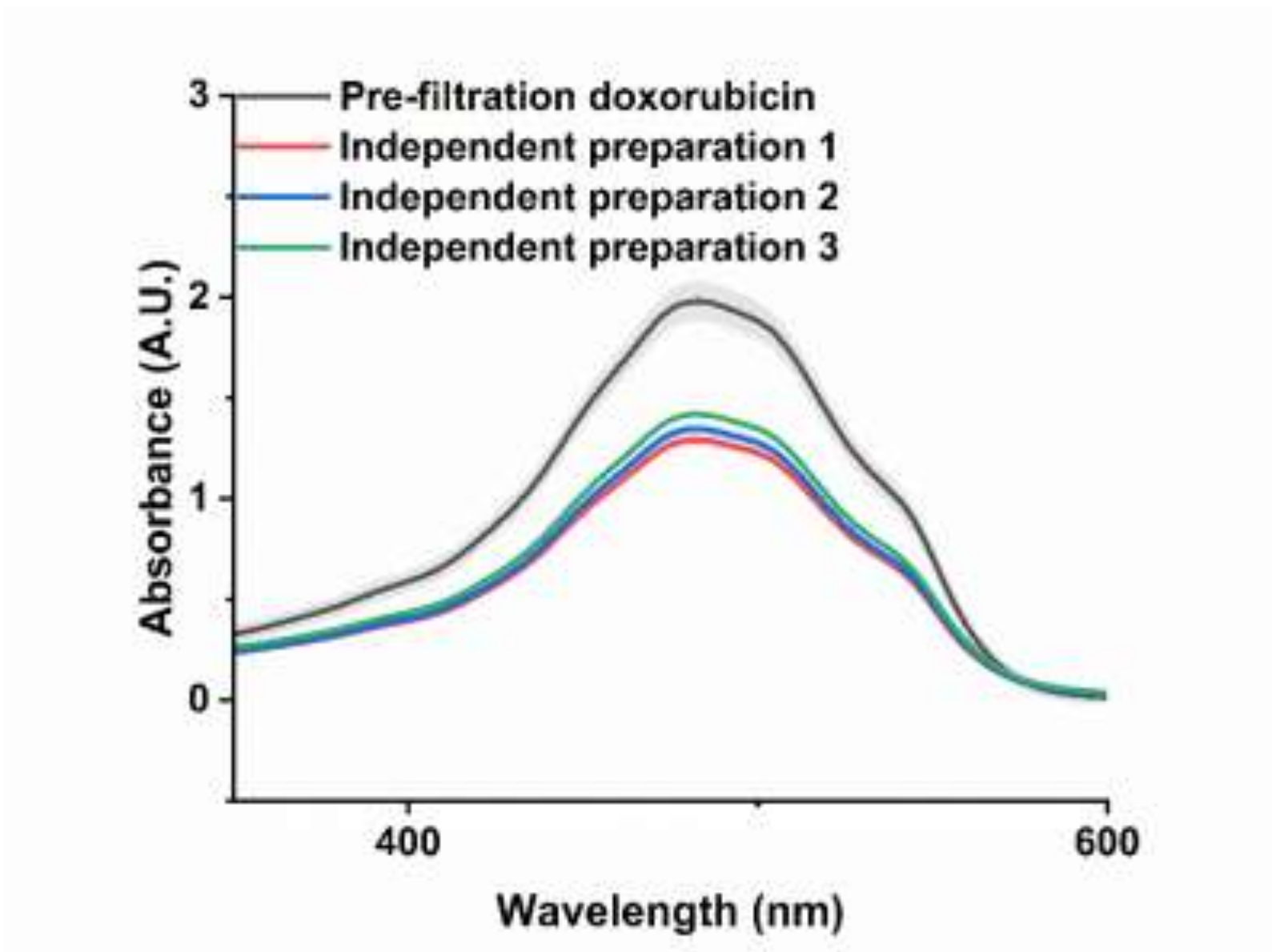

Figure S4. UV-Vis analysis of pre- and post-ultrafiltration doxorubicin solutions used for determination of encapsulation efficiency and drug loading. The initial doxorubicin solution used for polymersome self-assembly was analyzed by UV-Vis spectroscopy following a 1:2 (v/v, doxorubicin:water) dilution. Following self-assembly, unencapsulated doxorubicin was separated from the polymersomes by centrifugal ultrafiltration. The resulting filtrates were collected, dried, and resuspended to the original aqueous volume used during polymersome preparation (350 µL). The resuspended samples were then diluted 1:2 (v/v, sample:water) and analyzed by UV-Vis spectroscopy. Doxorubicin concentrations were determined using the standard curve shown in **Figure S3**. Encapsulation efficiency was calculated from the initial and filtrate doxorubicin concentrations using Equation 1 in the main manuscript, yielding an encapsulation efficiency of 32.4%. The encapsulated doxorubicin mass was then calculated and used to determine the drug loading percentage according to Equation 2, using 0.30 mg PBd-*b*-PEO, 0.35 mg AuNPs, and the calculated mass of encapsulated doxorubicin as the total carrier mass, correlating to a drug loading efficiency of 14.2%.

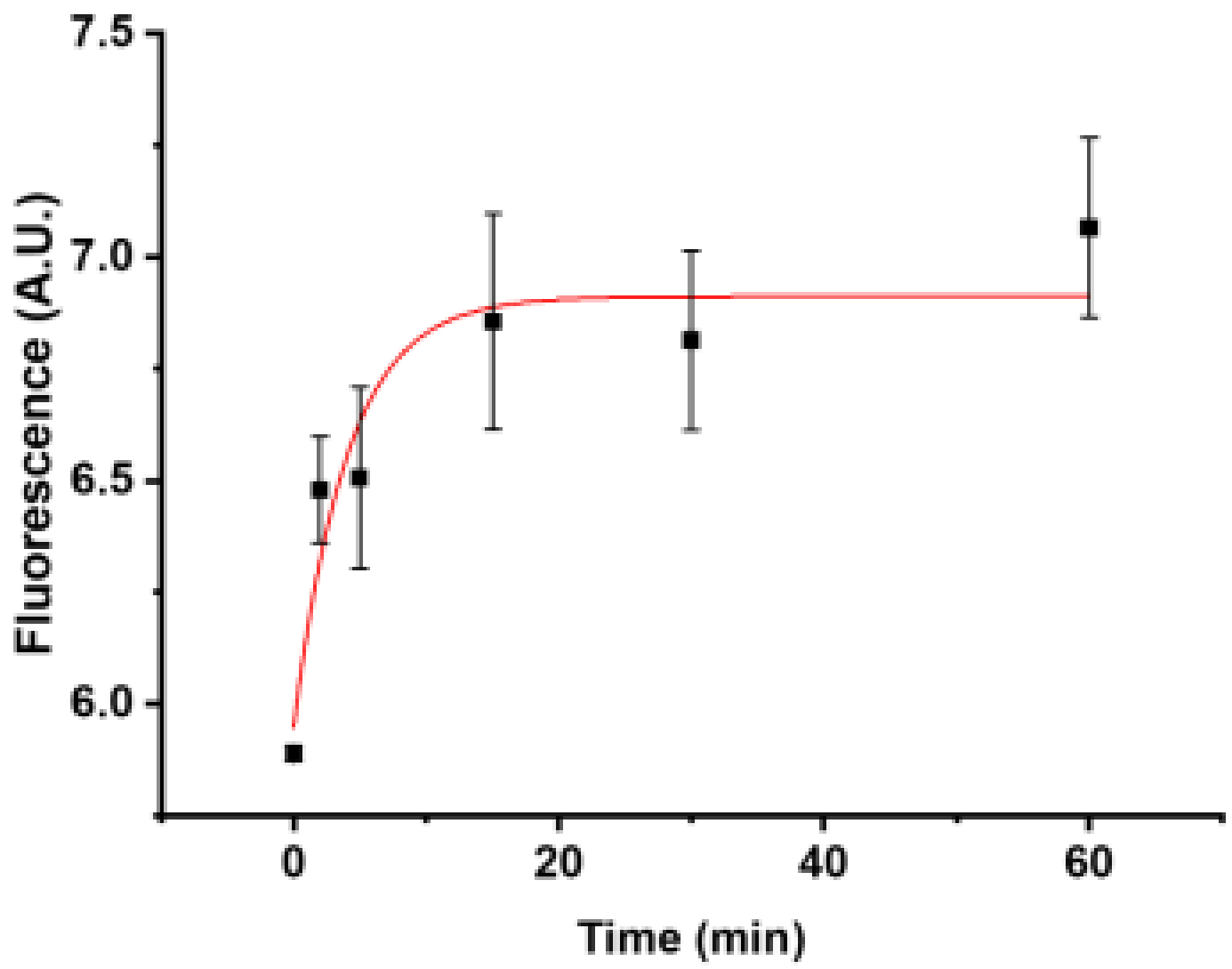

Figure S5. Uptake kinetics of Nile Red-loaded AuNP-polymersomes in NIH-3T3 cells. Cells were incubated with Nile Red-loaded AuNP-polymersomes for the indicated time points before extracellular polymersomes were removed by washing and the cells were fixed with formaldehyde. The fixed cells were imaged with fluorescence microscopy and the resulting images were analyzed to determine the mean fluorescence intensity per cell. The resulting uptake profile was fit using a one-phase association model (R^2= 0.90), yielding a rate constant of k = 0.245 ± 0.114 min^{-1} and an uptake half-time ($t_{1/2}$) of 2.8 min. Fluorescence intensity approached a plateau after approximately 15 min, indicating rapid intracellular polymersome accumulation.

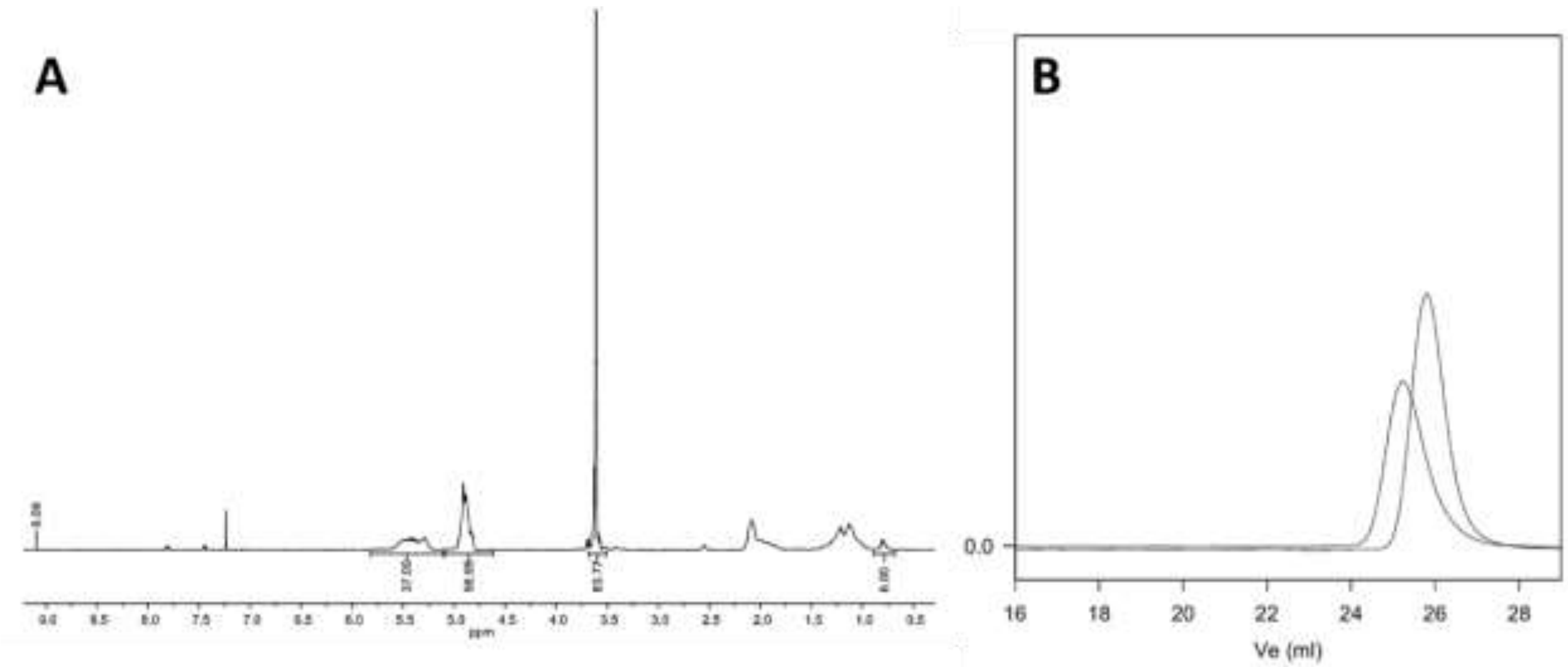

Figure S6. Characterization of the PBd-*b*-PEO polymer (Polymer Source, P43223B1-BdEO). (A) ^{1}H NMR spectrum of the PBd-*b*-PEO polymer. (B) Size exclusion chromatogram of the PBd-*b*-PEO polymer. ^{1}H NMR and SEC are reproduced from the product data sheet with permission from Polymer Source.
